# Genome Organization and Dynamics Specialty Grand Challenge

**DOI:** 10.3389/fmolb.2021.818707

**Published:** 2021-12-31

**Authors:** Brian Christopher Freeman

**Affiliations:** University of Illinois at Urbana-Champaign, Champaign, IL, United States

**Keywords:** genome organisation, transcription, chromatin, DNA repair, DNA replication, telomeres, nucleoskeleton

## Introduction

In encoding the fundamental information to create a cognate cell, genomes are often referred to as the blueprints of life. Yet, a perfunctory consideration of a common blueprint only envisions a flat almost lifeless paper. In reality, blueprints are lively conduits of work with the paper being rolled, folded, and even crumpled while the contents are continuously read, revised, and expanded to guide the construction of structures with balance and beauty. Comparatively, genomes are continuously read, modified, and reworked to mediate cell fate decisions and support cell health throughout all life stages and physiological conditions. Given the central importance of genomes, it is imperative to carefully shield chromosomes to ensure self- and species-preservation, yet the DNA must also be readily accessible to allow on-demand use of the information. To balance these two critical features cells have developed highly efficient systems to read, replicate, repair, and store genomic information. Intriguingly, despite decades of effort, we have only scratched the surface of our comprehension on how each system works as well as understanding how the various pathways intercommunicate to function in harmony and ensure homeostasis. The objective of the *Genome Organization and Dynamics* section of *Frontiers in Molecular Biosciences* is to provide a central forum for discussing and distributing high-quality scientific observations, concepts, and philosophies to all areas related to genome function and preservation.

*Genome Organization and Dynamics* has interests in highlighting all nuclear processes/pathways including **chromatin**, **transcription**, **DNA repair**, **telomeres**, **DNA replication**, **3D chromosome organization**, and the **nucleoskeleton** under both normal and disease conditions. We also welcome articles exploring the cutting-edge tools that are providing unprecedented insights into the various genome-associated pathways including physical and computational tactics based on molecular, biochemical, or cell biological assays focusing on single or bulk cell populations. In addition, we are open to serving as a platform to debate emerging concepts within the various fields of study. For further guidance, a few vignettes of exemplary topics are provided below.

## Transcription

Transcription is the process of converting the information encoded in a strand of DNA into RNA. While it was popularized by the central dogma of molecular biology describing the flow of genetic information as “DNA makes RNA and RNA makes protein” (Crick, 1958), it is now clear that the act of transcription, transcription factors/cofactors, and the produced RNAs serve a variety of roles within a cell beyond the creation of proteins (Kim and Shendure, 2019; Zaret 2020; Kolathur 2021). While new discoveries are being continuously reported within the field that are shaping the way we understand almost all facets of biology, classic questions remain unanswered despite decades of intensive scrutiny.

A deceivingly simple question that remains unresolved is determining how each transcription factor finds its cognate DNA element in a physiologically timely and useful manner. It has long been appreciated that activated transcription factors, even ones residing in the cytoplasm when quiescent, only require a few minutes to engage the appropriate response elements to drive a productive gene program (Freeman and Yamamoto 2001). Yet, how these proteins scan the millions of bases of DNA and select the correct sites remains an enigma. A favored model to help resolve the dilemma posits that transcription factors engage chromosomes at any site then slide along its length scanning for its cognate DNA element thereby turning a 3D exploration into a 1D search (Berg et al., 1981). While such 1D sliding has been observed for DNA repair proteins (Blainey et al., 2006; Gorman et al., 2010), comparable supportive evidence for any transcription factor is missing. Moreover, how any DNA binding protein might effectively “slide” through compacted heterochromatin is not apparent yet select sites within heterochromatin are recognized by pioneering transcription factors to facilitate an opening of these structures (Zaret 2020). Hence, the mechanism(s) used to rapidly build productive transcription factor-DNA complexes remains unresolved. Recent speculation has suggested transcription factors may form and rely on liquid/liquid phase transitions to foster enrichment around select DNA regions (Hnisz et al., 2017). Yet, empirical evidence validating this concept hasn’t been reported.

Once a transcription factor engages an appropriate DNA element there are several potential outcomes including controlling gene promoter activity through proximal (upstream activating sequences) or distal (enhancer) sites, opening heterochromatic areas (pioneering factors), or mobilizing chromosome regions (genome reorganization) (Kim and Shendure, 2019; Zaret 2020; Peng et al., 2021). Intriguingly, individual transcription factors can perform any one or all of these functions. However, we do not fully understand the determinants that dictate which outcome will occur. Despite not knowing how the choice is made, strides are being made in delineating the downstream events at different sites. For instance, genome-wide analysis suggests that transcription factors make distinct contributions to transcription rates with proximal elements primarily influencing transcription burst size and enhancers modulating burst frequency (Larsson et al., 2019). Why the proximity of the element leads to differential effects is not clear. Perhaps it is a matter of differential cofactor recruitment? Yet, not all transcription rates are governed in this manner. Single cell imaging work has shown that transcription factor-DNA dynamics can dictate burst size and that the bound fraction determines burst frequency at proximal elements (Stavreva et al., 2019). Thus, it is important to consider both ensemble approaches, which provide knowledge on the dominant mechanism of a particular process, and to consider single cell/molecule tactics, which give valuable insights on select targets to fully understand a system.

In addition to the described topics, *Genome Organization and Dynamics* is interested in exploring all facets of the transcription process—from the cellular roles of non-coding RNAs to the mechanisms of RNA polymerase movements (e.g., initiation, pausing, release, termination, etc.) to the involvement of nuclear bodies (e.g., speckles). Beyond transcription, we will also feature articles exploring how transcription intersects with other pathways working in the nucleus under normal and disease conditions.

## Chromatin

The genomes of most, if not all, organisms across the kingdoms of life are compacted and organized into chromatin through the actions of select factors including histones/histone-like proteins and non-coding RNAs (Dame et al., 2020; Sivakumar et al., 2019). Initial reports on chromatin structures were made by Emil Heitz in 1928 using a DNA-staining procedure that visualized densely (heterochromatin) and lightly stained (euchromatin) areas (Heitz, 1928), which correspond to nucleosome rich and sparse regions of chromosomes. Modern interests related to chromatin grew from the simple question of how to fit nearly 2 m of chromosomal DNA into the relatively tiny nucleus of a human cell (Alberts et al., 2002). We now appreciate that chromatin serves as a central constituent of most nuclear activities since it both influences the actions of DNA-associated processes and is also modified by the pathways that work along its surface. Hence, it is critical to gain a better understanding of chromatin biology.

*Genome Organization and Dynamics* is eager to highlight work on chromatin features of all organisms from bacteria to archaea to plants and animals. It is well-known that eukaryotes use nucleosomes as the basic structural units to package DNA into chromatin (Alberts et al., 2002). In a context dependent manner [*e.g*., organism/cell type, chromosome position (promoter, centrosome, damaged DNA, *etc*.), or physiological status (stress, aging, disease, *etc*.)], the histone proteins are biochemically modified to mark nucleosomes thereby triggering the recruitment/retention of select proteins to perform requisite work. Basically, the sequential modification of a nucleosome serves as a breadcrumb trail guiding the nucleation of specific proteins in a set order at a precise location. The most frequently considered variations to nucleosomes are post-translational modifications (e.g., acetylation, methylation, phosphorylation, ubiquitination, etc.) of the histone tails commonly referred to as the histone code (Jenuwein and Allis 2001). The study of the histone code and its system of protein “writers, readers, and erasers” have provided profound insights into numerous nuclear pathways and biological phenotypes since the chemical changes can serve as epigenetic marks resulting in genetically heritable traits (Jenuwein and Allis 2001). In addition to the histone code, nucleosomes can be altered using noncanonical histones such as H2A.Z, H2A.X, CENP-A (Talbert and Henikoff 2021). While variants can have clear roles within the nucleus (e.g., CENP-A is an epigenetic mark of centromeres), the purpose of H2A.Z is less well defined.

H2A.Z is considered a universal histone variant as it is found in most eukaryotic cells, has a highly conserved amino acid sequence, is relatively abundant (i.e., ∼5–10% of all nucleosomes contain it), and is essential in most eukaryotes (budding yeast is an exception) (Redon et al., 2002; Giaimo et al., 2019). H2A.Z is associated with gene promoters particularly with the +1 nucleosome (Giaimo et al., 2019). In budding yeast, H2A.Z marks the 5’ end of genes regardless of promoter activity and deletion of H2A.Z only mildly impacts global transcript levels in yeast (Raisner et al., 2005). In contrast, loss of H2A.Z in flies and mammals correlates with significant changes in transcription except in post-mitotic cells where H2A.Z disruption has little impact on promoter activity (Belotti et al., 2020; Lamaa et al., 2020; Scacchetti et al., 2020). How H2A.Z influences transcription in mitotic cells remains an open question. Does H2A.Z function as a roadblock to RNA polymerase II or does it foster entry of polymerase into a gene body (Zhang et al., 2004; Chen et al., 2019; Mylonas et al., 2021)? Alternatively, does H2A.Z modulate nuclear processes by facilitating repressive heterochromatin regions (Swaminathan et al., 2005; Wang et al., 2018; Courtney et al., 2020) or as a marker used to organize genomes (Oomen et al., 2019; Wang et al., 2020)? Hence, there is still much to be learned regarding the many layers of H2A.Z properties and function.

Epigenetic features from the histone code to histone variants are just a few of the chromatin-related topics that interest *Genome Organization and Dynamics*. We are pleased to consider all areas of chromatin biology including how histone-like proteins influence bacterial biofilms, the impact of chromosome inactivation on sex determination, or the role of heterochromatin in the aging process just to name a few.

## Topological Genome Organization

How genetic material within a cell is organized has long been studied (Rabl, 1885). While early electron micrographs supported the contention that chromosomes from bacteria to humans are differentially compacted within a cell (Alberts et al., 2002), it wasn’t until the development of advanced microscopy and molecular techniques that we were able to visualize the intricate organization of all genomes. Groundbreaking advances in super resolution microscopy have enabled unprecedented views of genomes within the three-dimensional space of whole cells leading to the discovery of chromosome territories and the monitoring of directed, long-distance chromatin movements within live interphase cells (Shaban et al., 2020). Novel molecular approaches, especially techniques based on the chromatin conformation capture (3C) assay, have validated the concept of select chromosome packaging and extended our resolution of the organization to the single base pair level (Dekker and Misteli 2015). The molecular assays have provided deep insights into which DNA sites are in close proximity to others or to select nuclear landmarks (e.g., nucleolus, nuclear envelope, etc.) thereby leading to numerous new discoveries including detailed cell type-specific genome organization, the existence of topologically associated domains (TADs), and lamina associated domains (LADs) (Dekker and Misteli 2015).

Although the phenomenon of precise 3D genome packaging is now well established (Dekker and Misteli 2015), how such organization is achieved within any organism is still unresolved (Peng et al., 2021). Minimally, large-scale features of the packaging correlate with the histone code (Pierro et al., 2017) but how nucleosome marks direct the precise positioning of an entire genome has not been shown. A prevailing model suggests that protein readers of the histone code (e.g., Heterochromatin Protein 1 or HP1) undergo physical phase transitions once bound to the chromatin to drive the organization (Larson et al., 2017). Yet, where phase transitioning proteins might gain the energy to guide/drive the folding of entire chromosomes isn’t apparent. Perhaps features of the genome/epigenome engage the nucleoskeleton system to achieve efficient mobility (Wang et al., 2020). Nevertheless, it is clear that certain factors, including cohesin and CCCTC-binding factor (CTCF), demark the boundaries of smaller features of genome organization including TADs and chromatin loops (Dekker and Misteli 2015). The physiological roles of these structures, however, are yet to be defined, as disruption of TADs by depletion of cohesin or CTCF only have limited influences on gene expression (Nora et al., 2017; Rao et al., 2014; Wutz et al., 2020). Hence, there is much to be understood regarding genome organization including how it is achieved and the physiological relevance for it.
